# Research on gut microbiota characteristics of PBC patients at different ALBI grades based on machine learning

**DOI:** 10.3389/fmicb.2024.1495170

**Published:** 2024-11-06

**Authors:** Han Shi, Qi Wang, Bin Xu, Yanmin Liu, Juan Zhao, Xue Yang, Chunyang Huang, Ronghua Jin

**Affiliations:** ^1^Beijing Ditan Hospital, Capital Medical University, Beijing, China; ^2^Key Laboratory of Emerging Infectious Diseases, Institute of Infectious Diseases, Bejing Ditan Hospital, Capital Medical University, Beijing, China; ^3^Beijing Institute of Infectious Diseases, Beijing, China; ^4^National Center for Infectious Diseases, Beijing Ditan Hospital, Capital Medical University, Beijing, China; ^5^Beijing You'an Hospital, Capital Medical University, Beijing, China; ^6^Changping Laboratory, Beijing, China

**Keywords:** primary biliary cholangitis, albumin-bilirubin, gut microbiota, lachnospira, machine learning

## Abstract

**Background:**

The Albumin-Bilirubin (ALBI) score and grade are widely used to stratify patients with primary biliary cholangitis (PBC) into different disease statuses and risk levels. Recent studies have increasingly highlighted the role of gut microbiota in autoimmune liver diseases. This study aimed to investigate the differences in gut microbiota among PBC patients with varying ALBI grades.

**Methods:**

Clinical data and stool samples were collected from outpatient and inpatient PBC patients between 2019 and 2022. Gut microbiota profiles were obtained using 16S rDNA sequencing of stool samples. We analyzed alpha diversity, beta diversity, LEfSe analysis and pathway function prediction. Additionally, various machine learning methods—including random forest (RF), lasso, gradient boosting machine (GBM) and support vector machine (SVM)—were employed to identify key features and to build and validate predictive models using bootstrap techniques.

**Results:**

Clinical characteristics of ALBI grade 1 patients were comparatively better than those of ALBI grade 2 and 3 patients, including multiple laboratory indices. Gut microbiota analysis revealed that species richness and balance were higher in ALBI grade 1 patients. Both the comparison of the most abundant genera and the linear discriminant analysis (LDA) in LEfSe demonstrated that *Lachnospira* had a higher abundance and better discriminative ability in ALBI grade 1. Pathway function prediction indicated that sulfur metabolism was upregulated in higher ALBI grades. Furthermore, RF identified 10 specific genera, which were then used to build and validate models for discriminating PBC patients according to their ALBI grades. All three models, developed using different machine learning methods, demonstrated good discrimination ability (mean AUC 0.75–0.80).

**Conclusion:**

This study highlights significant differences in gut microbiota profiles among PBC patients with different ALBI grades. The increased abundance of Lachnospira and upregulation of sulfur metabolism pathways are notable in patients with lower ALBI grades. The machine learning models developed based on gut microbiota features offer promising tools for discriminating between PBC patients with varying disease severities, which could enhance the precision of treatment strategies.

## Introduction

Primary biliary cholangitis (PBC) is a chronic autoimmune liver disease, where patients may not exhibit specific signs or symptoms in early stages. As the disease progresses, they often develop nonspecific signs or symptoms such as pruritus, fatigue, and jaundice. The most common abnormalities in liver function test are elevated alkaline phosphatase (ALP) and gamma-glutamyl transferase (GGT) (Hirschfield et al., 2017; Lindor et al., 2019). Since the pathogenesis of PBC remains unclear, involving genetic background, autoimmunity, and environmental factors, the onset and progression of PBC also exhibit heterogeneity. Given the heterogeneous nature of PBC progression among individuals, accurate patient classification and assessment are of crucial. In addition to conventional criteria like Paris-I, Paris-II, and Rotterdam, some studies have proven that albumin-bilirubin (ALBI) score at baseline could evaluate prognosis effectively. ALBI score was firstly raised in prognosis study of hepatocellular carcinoma. In PBC, higher ALBI score usually associated with higher liver-transplant rate and death risk (Xu et al., 2024; Chan et al., 2015; Yamashita et al., 2023).

The gut-liver axis plays a vital role in liver disease, encompassing complex interactions between the gut and liver. This involves not only their close anatomical relationship but also the exchange of various substances, including bile acids, gut microbiota, and microbial metabolites, which can influence the physiological status of both organs (Tilg et al., 2022; Li et al., 2017; Kummen and Hov, 2019). Previous research on non-alcoholic fatty liver disease (NAFLD) and alcoholic liver disease (ALD) have demonstrated that dysbiosis occurs in early stages of liver diseases and worsens as the disease progresses. In turn, this dysbiosis can increase gut mucosal permeability, leading to translocation of bacteria and subsequent chronic liver inflammation and fibrosis (Mao et al., 2015; Zhang et al., 2023). Recent studies on PBC patients have begun to uncover their unique gut microbiota profiles. Tang et al. demonstrated that gut microbiota profile of treatment-naïve PBC patients differed significantly from that of healthy controls. Their analysis showed that PBC-enriched genera, such as *Haemophilus*, *Streptococcus*, and *Pseudomonas*, decreased after treatment, while genera enriched in healthy controls, like *Bacteriodetes*, *Sutterella*, and *Oscillospira*, increased (Tang et al., 2018). These findings shed light on the study of PBC gut microbiota discovery.

In recent years, machine learning has been increasingly applied in gut microbiota research. Due to its non-invasive nature, this method is being used to enhance the classification capabilities of gut microbiota in order to evaluate liver disease progression (e.g., fibrosis and cirrhosis) and predict disease outcomes, with promising results (Liu et al., 2023). Therefore, we aimed to use machine learning techniques to analyze gut microbiota characteristics of PBC patients across different ALBI grades.

## Methods

### Patients’ data collection

We collected laboratory examination data from 75 patients with PBC who were initially diagnosed at Youan Hospital, Capital Medical University between 2019 and 2022, including blood routine tests, coagulation function tests, and liver function tests. The diagnostic criteria for PBC were based on the 2017 EASL guidelines for PBC diagnosis: (1) Abnormal elevation of ALP and/or GGT; (2) Positive anti-mitochondrial antibody; (3) Liver biopsy showing pathological changes associated with PBC (Hirschfield et al., 2017). Patients who met at least two of the above criteria were diagnosed with PBC. Exclusion criteria included: (1) Patients with viral hepatitis; (2) Patients with ALD, NAFLD, or drug-induced liver disease (DILI), unrelated to infection; (3) Patients with primary or metastatic liver cancer; (4) Patients who used antibiotics, probiotics, or prebiotics within the past 14 days; (5) Patients with intestinal diseases or who had undergone intestinal surgery; (6) Patients with severe heart or kidney dysfunction or other severe organic diseases.

The ALBI score is calculated as follows:


$$\begin{array}{l} \mathrm{ALBI score}=\left(0.66\times \log10\;\left(\mathrm{TBIL in}\;\mu \mathrm{mol}/L\right)\right)\\ -\left(0.085\times \mathrm{ALB}\;\mathrm{in}\;g/L\right) \end{array}$$

Based on the calculated ALBI score, patients were classified into three ALBI grades:

- ALBI Grade 1: ALBI score ≤ −2.60.
- ALBI Grade 2: ALBI score > −2.60 and ≤−1.39.
- ALBI Grade 3: ALBI score > −1.39 (Yamashita et al., 2023).

The present study was approved by the Ethics Committee of Capital Medical University affiliated Beijing You’an Hospital (No. LL-2019-128-K). All patients provided informed consent prior to participation.

### Stool collection and DNA extraction

All patients who provided stool samples ensured that they had not used antibiotics in the 14 days prior to collection. Stool samples from eligible patients were collected using sterile containers, promptly stored at −80°C, and transported under cold chain conditions. DNA from the samples was extracted using the MagPure Stool DNA KF Kit B (MAGEN). The integrity of the DNA was confirmed through 1.2% agarose gel electrophoresis, and its concentration and purity were verified.

The V3 and V4 regions of the bacterial 16S rDNA gene were amplified by polymerase chain reaction (PCR) using bacterial primers (338F 5′-ACTCCTACGGGAGGCAGCAG-3′ and 806R 5′-GGACTACHVGGGTWTCTAAT-3′).

### 16S rDNA sequencing and data analysis

Using the Circularization Kit User (MGIEASY), the circularization reaction system was prepared to obtain single-stranded circular products, and un-circularized linear DNA molecules were digested. The single-stranded circular DNA molecules were then subjected to rolling circle amplification to form DNA nanoballs (DNBs) containing multiple copies of the DNA sequence. The resulting DNBs were applied to the wells on a high-density DNA nanochip using the combined probe-anchor polymerization technique (cPAS) for sequencing [Sequencer: MGISEQ-2000, MGISEQ-2000RS High-throughput Rapid Sequencing Reagent Kit (FCS PE300)]. The sequencing data were subjected to quality control to obtain clean data.

The sequences were assembled using Fast Length Adjustment of Short reads (FLASH, v1.2.11) software, where paired-end reads generated by sequencing were assembled into a single sequence based on their overlapping regions, producing tags of the hypervariable regions. The assembled tags were then clustered into Operational Taxonomic Units (OTUs) based on 97% similarity using USEARCH (v7.0.1090) software. After obtaining the representative OTU sequences, taxonomic annotation was performed by comparing the OTU representative sequences with the database using the RDP classifier software, with a confidence threshold set at 0.6. Finally, OTUs with no annotation results or results not related to the analysis project were removed.

Alpha diversity was calculated using the specialized software package mothur (v.1.31.2) to assess the richness and evenness of the microbial communities, including indices such as the Chao1 index, ACE index, Shannon index, and Simpson index. Beta diversity analysis was performed using QIIME (v1.80) to evaluate the differences in species composition between different samples. This type of analysis is typically based on distance matrices; in this study, Bray-Curtis distance was used to measure the similarity or dissimilarity of species composition between samples. The phylogenetic characteristics of the gut microbiota and fecal microbial communities were analyzed using Linear Discriminant Analysis (LDA) and Linear Discriminant Analysis Effect Size (LEfSe) analysis.^1^ Additionally, microbial functional annotation was performed using PICRUSt2 (v2.3.0-b) based on the Kyoto Encyclopedia of Genes and Genomes (KEGG) database.

### Machine learning and statistical analysis

For continuous normally distributed variables, data are presented as mean ± standard deviation (SD) and analyzed using independent samples *t*-test. For continuous non-normally distributed variables, data are presented as median ± interquartile range (IQR) and analyzed using the Mann–Whitney U test. Categorical variables were analyzed using the chi-square test. The comparison of gut microbiota alpha diversity indices between the two groups was conducted using the Wilcoxon test.

For the machine learning analysis of patients’ gut microbiota, we used a bootstrap method (*n* = 100) for data feature selection and modeling. First, Random Forest was employed to calculate the Mean Decrease Accuracy and Mean Decrease Gini for the microbiota. The top 10 species identified by the intersection of these two importance measures were selected for subsequent modeling and validation. We used Lasso, Gradient Boosting Machine (GBM), and Support Vector Machine (SVM) methods for modeling. The receiver operating characteristic (ROC) curve was analyzed for each bootstrap iteration, and the area under the curve (AUC) values were summarized and plotted as a frequency histogram to assess the classification performance of the models. All statistical analyses and machine learning were performed using R (version 4.3.2). A *p* value <0.05 and FDR < 0.1 were considered statistically significant.

## Results

### Overall comparison of patients’ characteristics

Information from 75 PBC patients were collected. Based on ALBI calculation formula, patients were divided into ALBI grade 1 (ALBI_1) (*n* = 36), ALBI grade 2 (*n* = 36), and ALBI grade 3 (*n* = 3). Since there were only three patients in ALBI grade 3, we combined ALBI grade 2 and 3 (ALBI_2_3) to analyze the data. Overall characteristics of patients, including blood count, liver function test was compared in Table 1. Except bilirubin and albumin, which we used to classify the patients, white blood cells (WBC), platelets (PLT), prothrombin time (PT), prothrombin activity (PTA), international normalized ratio (INR), aspartate aminotransferase (AST), albumin/globulin (A/G), prealbumin (PALB), and total bile acid (TBA) all showed statistically significant differences between the two groups. The median level of WBC, PLT, PT, PTA, and INR were all within reference values. AST, A/G, PALB, and TBA all reflected that ALBI_1 was in relatively better status.

**Table 1:** Comparison of clinical characteristics of ALBI_1 and ALBI_2_3 patients.

		ALBI_1	ALBI_2_3	*p*
*n*		36	39	
Gender (%)	Female	35 (97.2)	34 (87.2)	0.202^1^
	Male	1 (2.8)	5 (12.8)	
Age (mean [SD])		50.26(11.93)	57.39(8.18)	0.018^2^
WBC (median [IQR])		5.65 [4.72, 6.72]	4.42 [3.19, 5.74]	0.005^3^
PLT (median [IQR])		235.00 [194.00, 271.00]	105.00 [73.00, 182.50]	<0.001
PT (median [IQR])		10.95 [10.60, 11.60]	11.90 [11.07, 14.55]	0.006
PTA (median [IQR])		104.00 [95.50, 110.00]	92.50 [63.25, 102.25]	0.004
INR (median [IQR])		0.96 [0.94, 1.03]	1.06 [1.00, 1.30]	<0.001
ALT (median [IQR])		35.50 [20.75, 60.75]	43.00 [18.00, 62.50]	0.535
AST (median [IQR])		36.00 [26.00, 67.00]	71.20 [44.50, 93.50]	0.006
TBIL (median [IQR])		18.20 [12.90, 21.55]	31.70 [19.25, 53.15]	<0.001
ALB (median [IQR])		45.20 [43.10, 47.02]	37.40 [33.20, 39.35]	<0.001
A/G (median [IQR])		1.27 [1.19, 1.52]	1.10 [0.86, 1.35]	0.001
GGT (median [IQR])		110.00 [34.05, 203.75]	154.00 [73.00, 310.50]	0.090
ALP (median [IQR])		174.00 [121.00, 241.40]	184.00 [134.50, 331.00]	0.645
PALB (median [IQR])		203.50 [169.25, 223.50]	125.00 [79.50, 152.25]	<0.001
TBA (median [IQR])		14.00 [7.78, 21.52]	57.10 [24.75, 106.45]	<0.001
CR (median [IQR])		49.00 [42.75, 51.75]	49.00 [44.00, 59.35]	0.393
TG (median [IQR])		1.29 [1.09, 1.68]	1.21 [0.88, 1.63]	0.436
CHOL (median [IQR])		5.56 [4.98, 6.55]	5.34 [4.18, 6.81]	0.216
HDL (median [IQR])		1.46 [1.21, 2.01]	1.25 [1.00, 1.77]	0.163
LDL (median [IQR])		3.31 [2.79, 3.82]	2.80 [2.18, 3.68]	0.129

1: Chi-square test; 2: independent samples *t*-test; 3: Mann–whitney U test. WBC, White blood cell; PLT, Platelet; PT, Prothrombin time; PTA, Prothrombin activity; INR, International normalized ratio; ALT, Alanine aminotransferase; AST, Aspartate aminotransferase; TBIL, Total bilirubin; ALB, Albumin; A/G, Albumin/globulin ratio; GGT, Gamma-glutamyltranspeptidase; ALP, Alkaline phosphatase; PALB, Pre-albumin; TBA, Total bile acids; CR, Creatinine; TG, Triglycerides; CHOL, Cholesterol; HDL, High-density lipoprotein; LDL, Low-density lipoprotein; ALBI, Albumin-bilirubin. Normal reference range: WBC 3.5 ~ 9.5 *10^9^/L; PLT 120 ~ 350 *10^9^/L; PT 9.9 ~ 12.8 s; PTA: 80 ~ 120%; INR: 0.8–1.2; ALT 7 ~ 40 U/L; AST 13 ~ 35 U/L; TBIL 5 ~ 21 μmol/L; ALB 40 ~ 55 g/L; A/G: 1.2 ~ 2.4; GGT 7 ~ 45 U/L; ALP 50 ~ 79 U/L; PALB: 200 ~ 400 mg/L; TBA: μmol/L; TG: <1.7 mmol/L; CHOL: <5.18 mmol/L; HDL: 1.0–1.6 mmol/L; LDL: <2.6 mmol/L.

### Analysis of gut microbiota information

**Figure 1:**
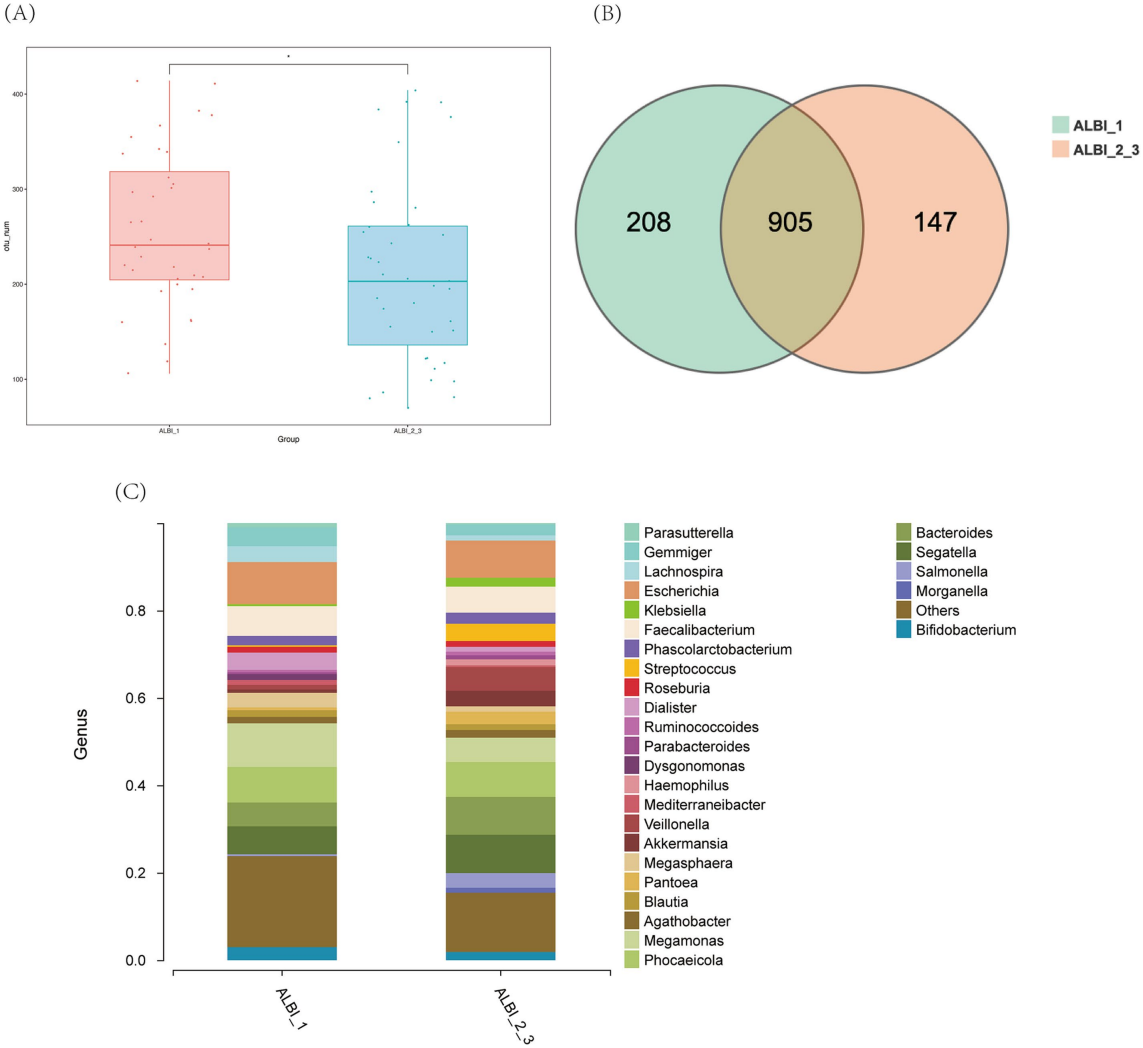
Overall characteristics of gut microbiota in different ALBI groups. **(A)** OTU number comparison of ALBI_1 and ALBI_2_3; **(B)** Venn diagram of OTU distribution in different groups; **(C)** The comparative distribution of the most prevalent genera across the two groups.

**Figure 2:**
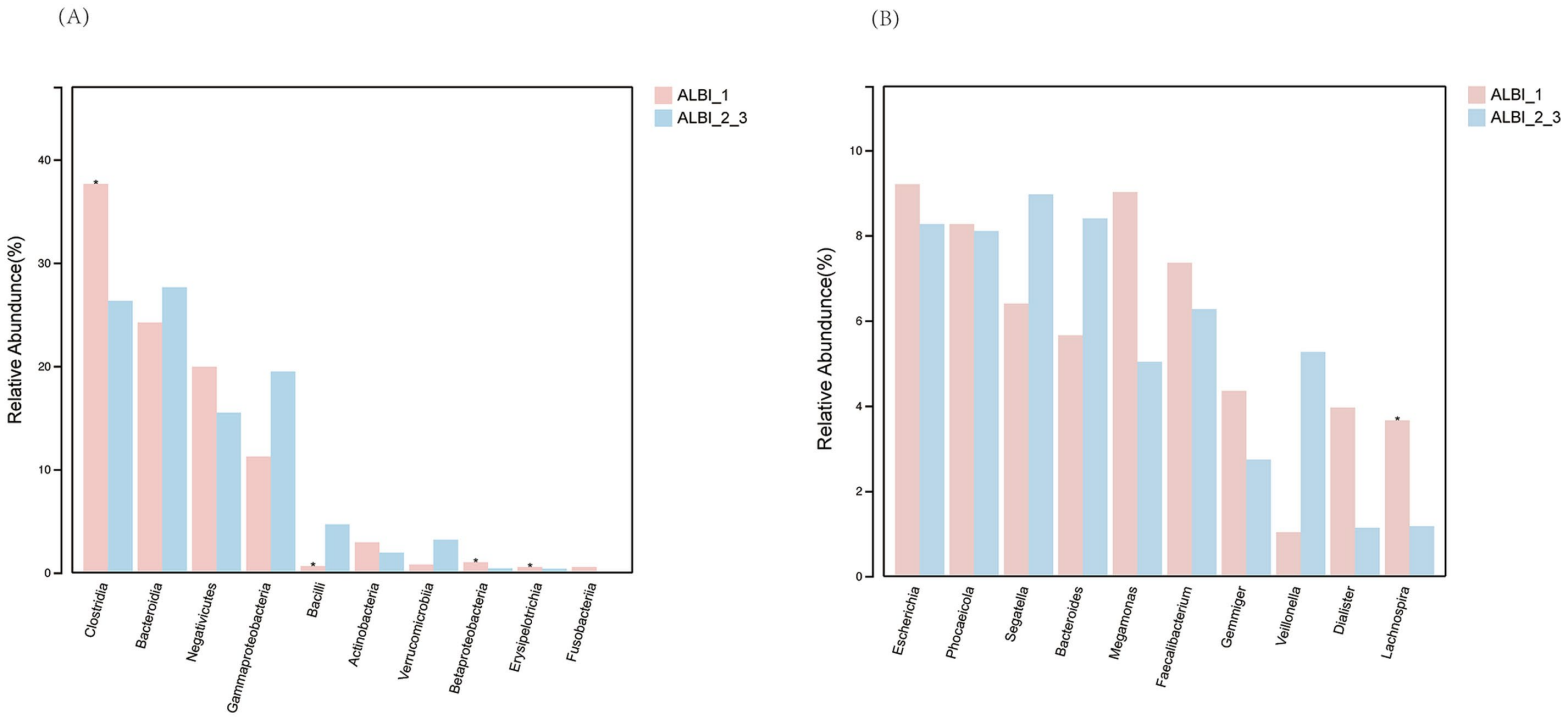
Relative abundance comparison of most abundant bacteria in different ALBI groups. **(A)** Class level; **(B)** Genus level.

Based on the results of 16S sequencing, we compared the microbial characteristics between ALBI_1 and ALBI_2_3. The comparison of Operational Taxonomic Units (OTUs) showed that the number of OTUs in the ALBI_2_3 group was significantly lower than that in the ALBI_1 group (*p* < 0.05) (Figure 1A). A Venn diagram analysis of the overlap of OTUs between samples indicated that ALBI_1 and ALBI_2_3 shared 905 species (Figure 1B). The result also revealed that gut microbiota profile differed between ALBI_1 and ALBI_2_3 groups. The most abundant top 10 bacteria in class level were *Clostridia, Bacteroidia, Negativicutes, Gammapropeobacteria, Bacilli, Actinobacteria, Verrucomicrobiia, Betaproteobacteria, Erysipelotrichia*, *and Fusobacteriia.* ALBI_1 showed significant expansion of *Clostrdia, Betaproteobacteria*, and *Erysipelotrichia* (*p* < 0.05); *Bacilli* exhibited significant decrease in ALBI_1 (*p* < 0.05), while other bacteria class did not demonstrated significant differences (Figure 2A). Analyzing the species composition at the genus level, the top 10 genera in relative abundance in both groups were *Bacteroides*, *Dialister*, *Escherichia*, *Faecalibacterium*, *Gemmiger*, *Megamonas*, *Phocaeicola*, *Segatella*, *Veillonella*, and *Lachnospira* (Figure 1C). A comparison of the top 10 genera between the two groups revealed that the relative abundance of *Lachnospira* was significantly higher in ALBI_1 than in ALBI_2_3 (*p* < 0.05), but other genera did not significantly enrich in either group. (Figure 2B). These results indicate that there are significant differences in the gut microbiota composition between PBC patients with different ALBI scores, suggesting that alterations in the gut microbiome may be closely associated with disease progression and liver function deterioration in PBC. The expansion of beneficial bacteria such as *Clostridia* and *Lachnospira* in the ALBI_1 group may be linked to maintaining better liver function.

**Figure 3:**
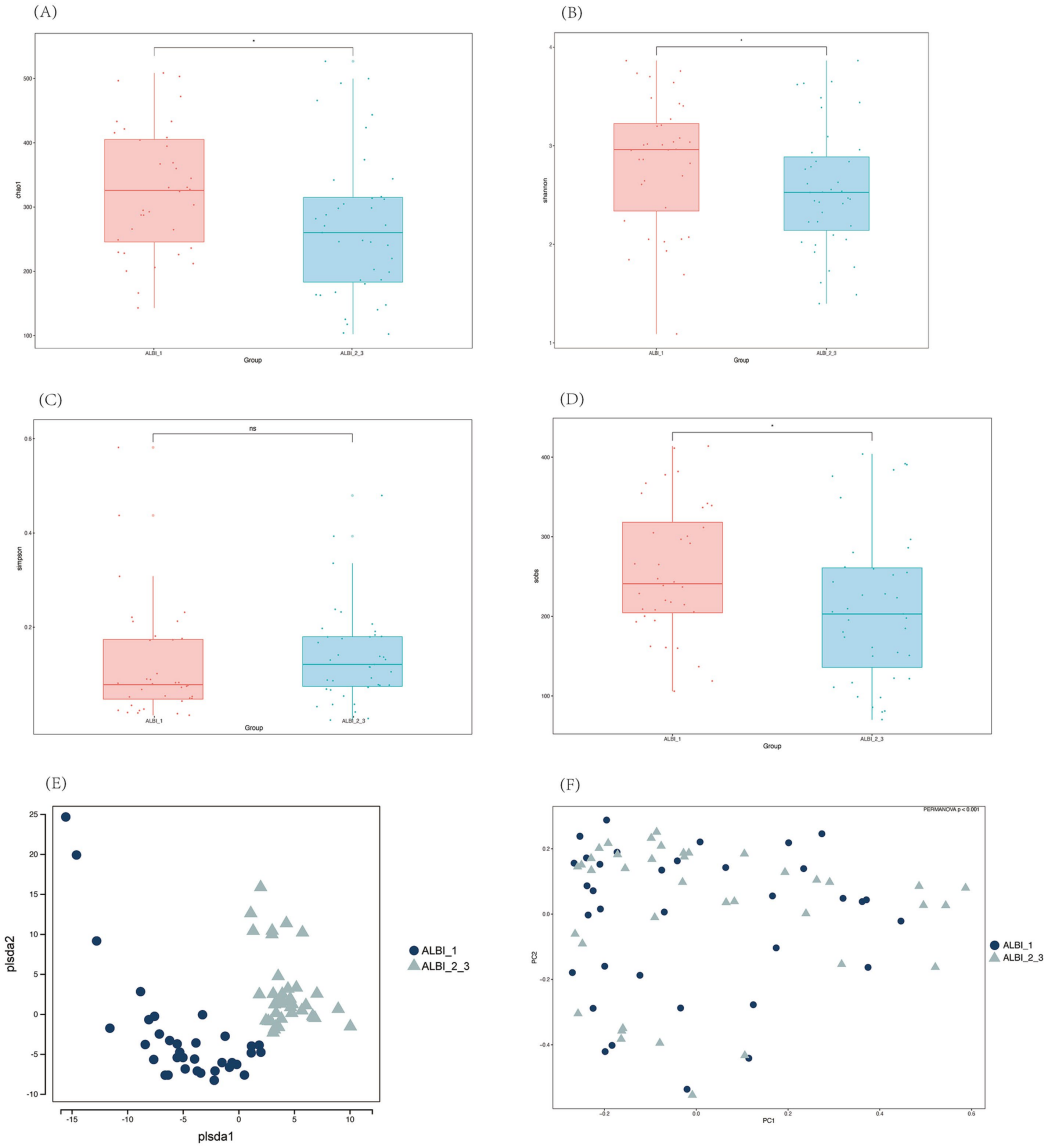
Alpha **(A–D)** and beta **(E,F)** diversity analysis among different ALBI groups. **(A)** Chao1 index; **(B)** Shannon index; **(C)** Simpson index; **(D)** Sobs index; **(E)** PLS-DA; **(F)** PCoA.

Alpha diversity analysis of the two groups showed that, except for the Simpson index, the Chao1, Shannon, and Sobs indices all exhibited significant differences between the two groups. These three indices were higher in the ALBI_1 group compared to the ALBI_2_3 group, indicating that the species richness and evenness were greater in ALBI_1 (Figures 3A–D). PLS-DA analysis and PCoA analysis based on the Bray-Curtis distance showed some differences in the distribution between the two groups (PERMANOVA *p* < 0.001) (Figures 3E,F).

### Phylogenetic characteristics of fecal microbiota in PBC patients with different ALBI grades

**Figure 4:**
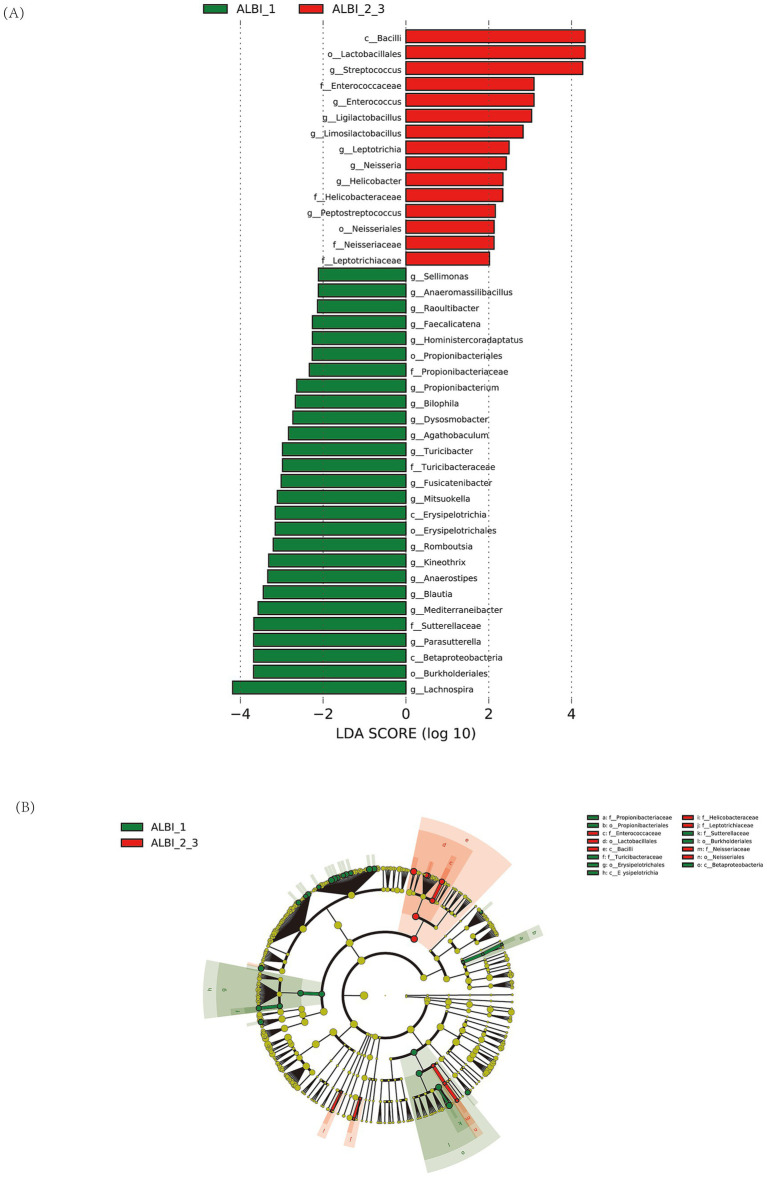
LDA analysis **(A)** and LEfSe cluster analysis **(B)** identified microbial taxa with significant discriminatory power between ALBI grade groups.

The results of the LEfSe LDA analysis show microbial taxa that have a significant impact in different ALBI grade groups (Figure 4A). In the ALBI_1 group, 27 species demonstrated significant discriminatory power (*p* < 0.05), with *g_Lachnospira* being the most prominent (LDA score > 4). In the ALBI_2_3 group, 15 species showed significant discriminatory power, with *c_Bacilli*, *o_Lactobacillales*, and *g_Streptococcus* having the highest scores (LDA >4). The LEfSe cladogram illustrates the significantly different microbial taxa, where the color of the nodes reflects their abundance in the corresponding group, and the hierarchical structure from phylum to genus is displayed from the inside out (Figure 4B). Combining the LDA results, the *c_Bacilli*, *f_Enterococcaceae*, and *o_Lactobacillales* taxa, which have significant discriminatory power in the ALBI_2_3 group, show a noticeable increase in abundance in the cladogram and form an overlapping cluster on the phylogenetic tree. This indicates that these taxa are closely related evolutionarily and are co-enriched in this group, possibly reflecting the adaptive advantage or functional relevance of this evolutionary branch in ALBI_2_3. It suggests that selective pressure in this specific group enhances the prominence of this branch within the microbial community.

### Functional analysis of gut microbiota differences in PBC patients with different ALBI grades

**Figure 5:**
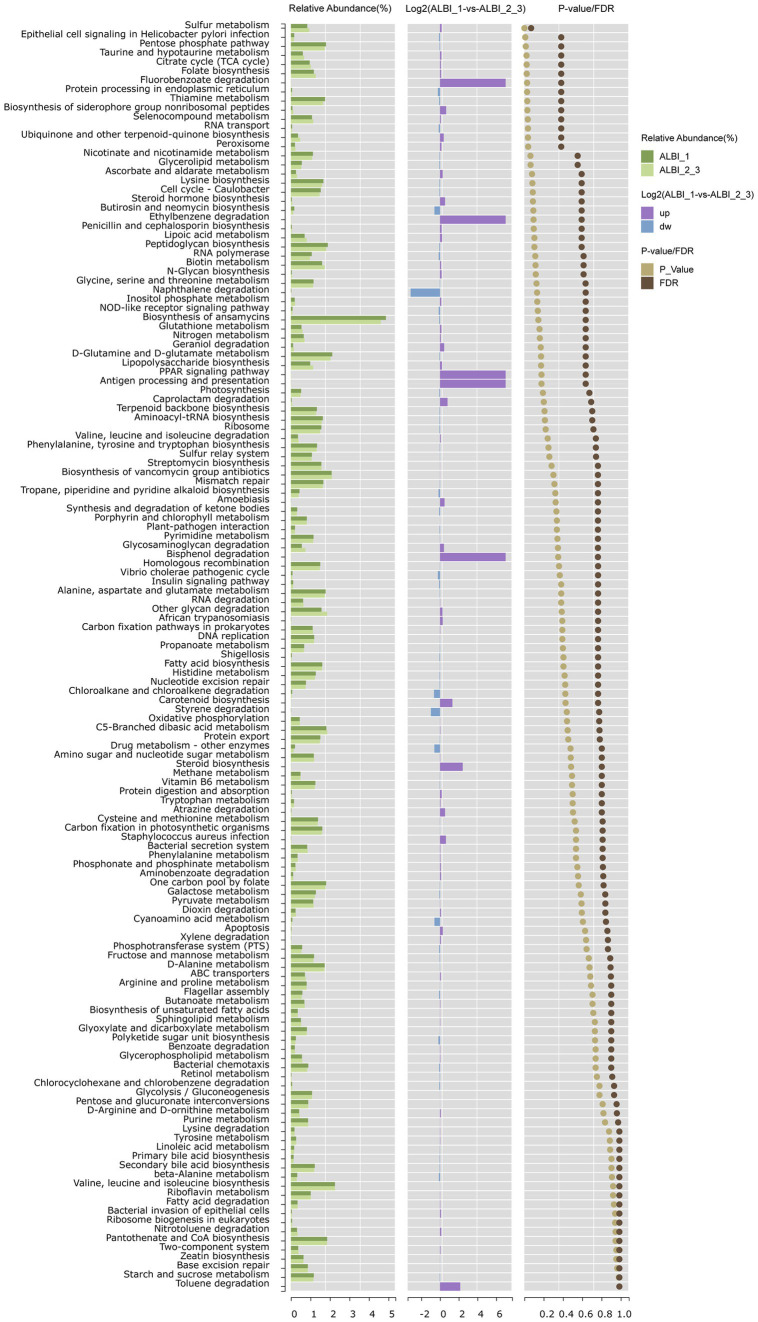
Pathway enrichment analysis comparing ALBI grade 1 and grades 2–3 groups. The chart shows the relative abundance, fold change (Log2), and significance (*p* value/FDR) of metabolic pathways between the two groups.

Using PICRUSt to predict microbial community gene functions based on sequencing data, it was found that pathways such as sulfur metabolism, pentose phosphate pathway, taurine and hypotaurine metabolism, citrate cycle, folate biosynthesis, thiamine metabolism, selenocompound metabolism, and ubiquinone and other terpenoid-quinone biosynthesis were relatively abundant in both groups. These pathways showed statistically significant differences between the two groups (*p* < 0.05). After FDR correction, sulfur metabolism exhibited the most pronounced difference between the two groups (*p* < 0.05, FDR < 0.1), with higher expression in the ALBI_1 (Log 2 ALBI_1 vs. ALBI_2_3 = 0.17) (Figure 5). In the human body, various organic compounds, such as carbohydrates, amino acids, and cholesterol derivatives, undergo sulfation and desulfation processes. The microbiome can regulate these processes through its own enzymes, thereby impacting human health.

### Machine learning classification analysis

**Figure 6:**
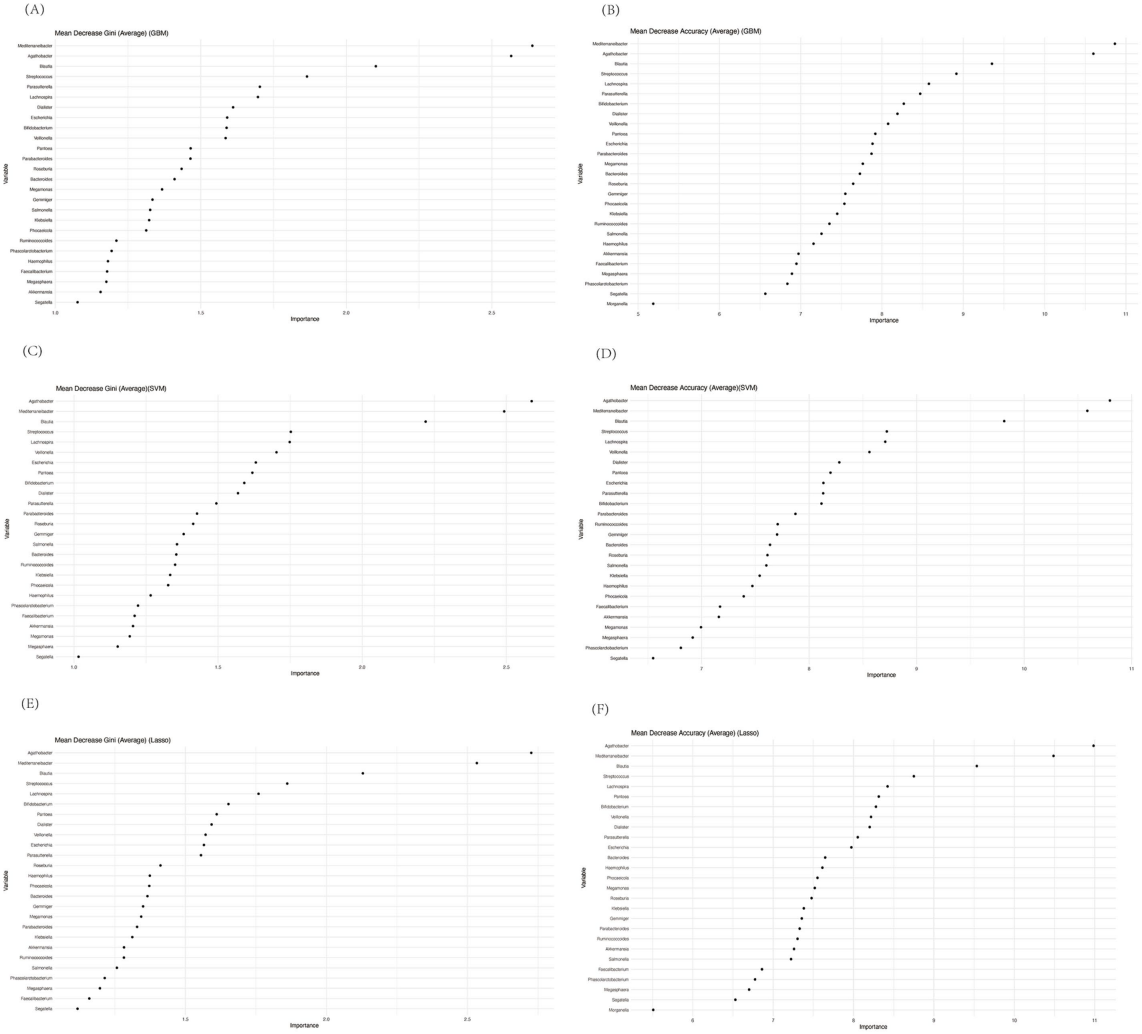
Mean decrease gini and mean decrease accuracy of genera calculated by random forest before performing GBM **(A,B)**, SVM **(C,D)**, or Lasso **(E,F)**.

As outlined in the methods section, we employed a bootstrap approach to enhance the robustness of our data analysis. Initially, we constructed a random forest model and calculated both Mean Decrease Accuracy and Mean Decrease Gini to rank the microbial taxa based on their importance in classification. Since we used bootstrapping, we repeated this process at each stage of the machine learning calculations, aggregating results across multiple iterations. The bootstrapped random forest models demonstrated consistent stability, ensuring the robustness of the selected features. Based on importance rankings from both Mean Decrease Accuracy and Mean Decrease Gini, we selected the top 10 species for further modeling and validation. These genera were *Mediterranebacter, Agathobacter, Blautia, Streptococcus, Lachnospira, Parasutterella, Bifidobacterium, Dialister, Veillonella, and Pantoea* (Figures 6A–F).

Notably, *Dialister, Lachnospira, and Veillonella* were among the most distinctive genera, aligning with previous studies that have highlighted their potential roles in gut-liver axis interactions and immune modulation. Interestingly, most of the other selected genera, such as *Mediterranebacter* and *Bifidobacterium*, were not the most abundant in our previous analysis, emphasizing that random forest tends to prioritize genera with high predictive value, rather than the highest abundance. This discrepancy highlights the utility of machine learning models in uncovering subtle but significant microbial patterns that may be missed using abundance-based analyses alone.

Next, we implemented three additional machine learning models: Gradient Boosting Machine (GBM), Support Vector Machine (SVM), and Lasso regression. To assess the predictive performance of each model, we employed a bootstrap-based validation strategy and calculated the Area Under the Curve (AUC) using ROC analysis. Across 100 bootstrap iterations, the models demonstrated consistent and reliable performance, as reflected in the mean AUC values. Specifically:

- GBM yielded an average AUC of 0.77 (sd = 0.04),
- SVM produced an average AUC of 0.76 (sd = 0.04), and
- Lasso resulted in an average AUC of 0.75 (sd = 0.05).

**Figure 7:**
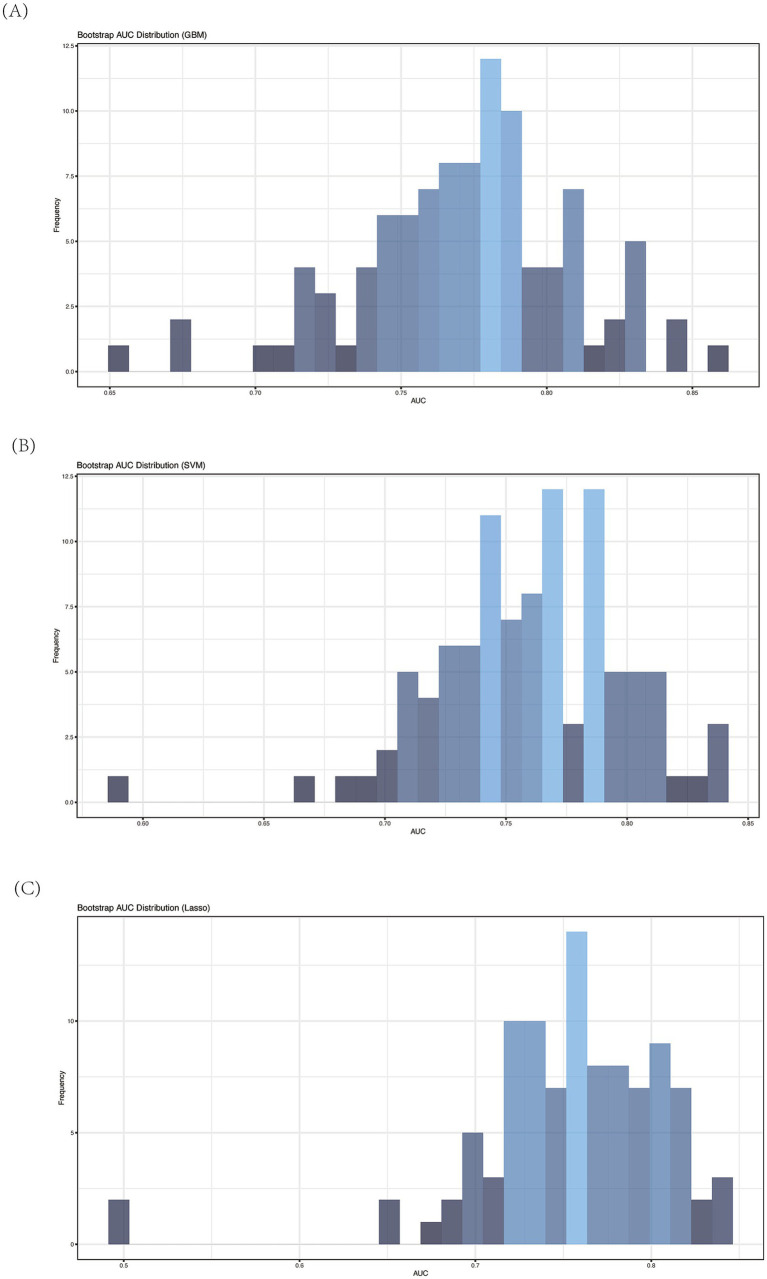
Average AUC of ROC analysis of different machine learning model by bootstrap (*n* = 100). **(A)** GBM; **(B)** SVM; **(C)** Lasso.

The consistency of these AUC values across different models indicates the robustness of the selected features for distinguishing between the two ALBI patient groups. Figures 7A–C presents the frequency distribution histograms of AUCs for each method, with the highest frequency of AUC values concentrated in the 0.75–0.80 range. These histograms further validate that the selected microbial taxa provide stable classification performance across different machine learning techniques.

## Discussion

The role of gut microbiota in the study of PBC is receiving increasing attention. Among the various criteria for predicting PBC prognosis, the recently introduced ALBI grade has shown significant potential, though it still requires validation from multiple perspectives. In our study, we compared PBC patients categorized by ALBI grade, providing a cross-sectional analysis of their clinical information along with the comparative characteristics of their gut microbiota, including functional pathway predictions. We then applied the random forest method to identify the 10 most distinguishing features between patients of different ALBI grades. The predictive power of these key features was further validated using GBM, SVM, and Lasso methods, all of which confirmed their utility in constructing reliable predictive models.

Our clinical data revealed significant differences in other prognostic indicators between the ALBI_1 and ALBI 2_3 groups, including TBIL and TBA levels, which were significantly lower in the ALBI_1 group. These findings align with previous studies, reinforcing that the ALBI grade correlates with the patient’s overall condition—where a lower ALBI grade is associated with better patient status and prognosis.

In terms of gut microbiota composition, our analysis revealed that the ALBI_1 group exhibited higher species richness and evenness (as indicated by Chao1, Shannon, and ACE indices) compared to the ALBI_2_3 group in alpha diversity analysis. Furthermore, both PCoA and PLS-DA analyses also demonstrated a significant difference in gut microbiota composition between the two groups. These findings suggest that patients in the ALBI_1 group, who are in a milder disease state, have a more balanced gut microbiota, which aligns with previous research. It has been established that species richness is generally lower in PBC patients compared to healthy controls (Tang et al., 2018; Furukawa et al., 2020). Wang et al. (2024) further found that species richness is reduced in PBC patients with cirrhosis compared to those without. The observed decrease in gut microbiota richness and diversity in ALBI_2_3 patients with poorer liver function may be explained by the disrupted gut-liver axis in advanced liver disease. As liver function deteriorates, factors such as bile acid composition, intestinal permeability, and immune responses are altered, leading to an imbalance in the gut microbiota. The reduction in beneficial microbial species and overall microbial diversity could further contribute to inflammation and metabolic dysregulation, exacerbating liver damage and promoting disease progression (Tilg et al., 2022; Hsu and Schnabl, 2023).

Interestingly, in the classical PBC microbiota study by Tang et al., no significant difference in diversity was observed when patients were classified based on their albumin and bilirubin levels (Tang et al., 2018). This discrepancy might be due to the heterogeneity in PBC progression among individuals or differences in methodology. While Tang et al. classified patients directly based on albumin and bilirubin levels, our study used a comprehensive calculation of these two indicators for classification. These studies collectively support the value of ALBI criteria in classifying gut microbiota in PBC patients.

We discovered the difference of the most abundant classes and genus bacteria of the two groups. Among these bacteria, Clostridia and *Lachnospira* was identified as the most discriminative species between the groups at genus level, with significantly higher relative abundance in the ALBI_1 group. *Lachnospira*, an anaerobic bacterium belonging to the *Clostridia* class within the *Firmicutes* phylum and the *Lachnospiraceae* family. The *Lachnospiraceae* family, to which *Lachnospira* belongs, is known for its ability to ferment carbohydrates in the host to produce various short-chain fatty acids (SCFAs), including acetate, propionate, and butyrate (Vacca et al., 2020). SCFAs have been shown to participate positively in liver disease in multiple ways. Tang et al. found that *Faecalibacterium*, another SCFA producer, decreases in PBC patients (Tang et al., 2018). In a study by Lammert et al., the relative abundance of *Lachnospiraceae* of PBC patients in late-stage fibrosis was significantly lower compared to those without, and stool acetate levels in non-late-stage were positively correlated with *Lachnospiraceae* group NK4A136 (Lammert et al., 2021). SCFAs can influence immune cell function by promoting the differentiation of dendritic cells and regulatory T cells (T regs), increasing immune tolerance (Zhang et al., 2023). Moreover, Wang et al.’s recent study showed that patients with poor UDCA response had decreased butyrate levels, and butyrate could improve cholangitis by inhibiting HDAC3 in myeloid-derived suppressor cells (MDSCs), enhancing acetylation of histone H3 lysine 27 (Wang et al., 2024).

Other studies have highlighted the protective role of SCFAs in liver disease. For instance, in a study on acute liver injury (AILI), fecal transplantation enriched for Lachnospiraceae and SCFAs promoted recovery of mitochondrial membrane potential, prevented ferroptosis, reduced DNA oxidation and lipid peroxidation, and alleviated AILI in mice (Yang et al., 2024). In NAFLD-related animal studies, feeding high-fat diet mice with sodium butyrate improved their liver biochemical levels, restored tight junctions in the small intestine, and reversed gut microbiota changes induced by a high-fat diet toward a profile similar to that of healthy controls (Zhou et al., 2017). Sodium butyrate can inhibit the progression of NAFLD by increasing histone acetylation and enhancing the sensitivity to glucagon-like peptide-1 (Zhou et al., 2018). In summary, these findings suggest the potential of SCFAs as research targets in the occurrence and progression of PBC.

In our pathway prediction using PICRUSt, sulfur metabolism emerged as the most significantly altered pathway. In living organisms, sulfur mainly exists as sulfur-containing amino acids (e.g., cysteine and methionine) and sulfates (Carbonero et al., 2012) Microbial sulfation and desulfation of carbohydrates have important effects on host health. Studies have shown that tyrosine can be metabolized into p-cresol sulfate through sulfur metabolism pathways in the body, which has been shown to alleviate inflammation in PBC by modulating the polarization of Kupffer cells and promoting the expression of anti-inflammatory factors (Fu et al., 2022). Additionally, reactive sulfur species (RSS), like H_2_S and H_2_S_2_, are generated in the process of sulfur metabolism, which can protect cells from oxidative stress (Xiao et al., 2018; Powell et al., 2018; Akaike et al., 2017). Uchiyama et al. demonstrated that gut microbiota could produce RSS and alleviate oxidative stress damage in Concanavalin A-induced mice model. *Lachnospiraceae* was found to produce high levels of RSS, which may explain why *Lachnospira* abundance and sulfur metabolism were both upregulated in ALBI_1 group (Uchiyama et al., 2022).

To more accurately assess whether gut microbiota-based models can classify patients by ALBI grade, we applied machine learning techniques to identify the most distinguishing microbial features. Using random forests for feature selection, followed by validation with GBM, SVM, and Lasso, we found that all three methods consistently produced AUC values within a narrow range (0.75–0.80). This consistency underscores the robust discriminatory ability of the selected microbial features.

Moreover, the random forest model’s emphasis on predictive value, rather than sheer abundance, allowed us to identify key genera that are potentially critical in PBC progression and ALBI classification, despite not being among the most abundant taxa. The stability and consistency of these features across multiple models reinforce the reliability of our approach. By leveraging machine learning, we can assist clinicians in identifying potential biomarkers for diagnosis, prognosis assessment, and disease monitoring, enabling more precise and effective handling of large, multidimensional data.

In our machine learning analysis, *Lachnospira* emerged as one of the top microbial features distinguishing between different ALBI grades. This finding not only reinforces the previously discussed higher abundance of *Lachnospira* in the ALBI_1 group, but also supports its biological significance in sulfur metabolism, as outlined earlier. While sulfur metabolism was identified through pathway analysis, *Lachnospira*’s selection by the machine learning model underscores its central role in both gut microbiota composition and metabolic function in PBC patients.

Thus, the machine learning model not only highlights *Lachnospira*’s discriminatory power, but also aligns with earlier observations linking its elevated abundance to upregulated sulfur metabolism, contributing to the anti-inflammatory and antioxidative processes observed in milder PBC cases (ALBI_1). These results across both biological pathway analysis and machine learning classification reinforces *Lachnospira’*s role as a key player in the gut-liver axis, further validating its relevance in PBC progression.

This study has several limitations that should be considered when interpreting the findings. First, the sample size was relatively small, particularly in the machine learning analysis. Although we used the bootstrap method to resample the data and address this issue, it may still introduce some risk of overfitting. To enhance the reliability of the results, future studies should involve larger, independent cohorts for validation.

Second, the study duration was relatively short. Given the chronic nature of PBC, longer study periods would allow us to observe more significant and long-term changes in gut microbiota composition and disease progression. Future research should aim to extend the study duration to capture these trends over time.

In addition to these primary limitations, the current sequencing depth may not have been sufficient to capture the full microbial diversity present in the samples. We recommend that future studies employ metagenomic approaches for more comprehensive sequencing, which could provide a deeper understanding of microbial functional pathways and interactions.

Despite these limitations, our study provides valuable insights into the gut microbiota profiles of PBC patients across different ALBI grades, and demonstrates the potential of machine learning techniques in identifying key microbial features for disease classification.

## Conclusion

In conclusion, we investigated the gut microbiota profiles of PBC patients with different ALBI grades, demonstrating the significance of ALBI classification from the perspective of gut microbiota. We found that *Lachnospira* was both discriminative in abundance and function by directly analyzing gut microbiota profile and machine learning methods. Besides *Lachnospira*, we also found other bacteria that function in distinguishing ALBI_1 and ALBI_2_3 PBC patients. In the future, combining ALBI classification with microbiota characteristics may enable more precise patient differentiation and prognostic assessment.

## Data Availability

Data to support the study findings are available on request from the corresponding author.

## Footnotes

^1^ https://huttenhower.sph.harvard.edu/galaxy/
